# Comparison of perioperative outcomes between robotic-assisted and video-assisted thoracoscopic surgery for mediastinal masses in patients with different body mass index ranges: A population-based study

**DOI:** 10.3389/fsurg.2022.963335

**Published:** 2022-07-14

**Authors:** Rongyang Li, Zheng Ma, Chenghao Qu, Jianhao Qiu, Kun Wang, Weiming Yue, Hui Tian

**Affiliations:** Department of Thoracic Surgery, Qilu Hospital of Shandong University, Jinan, China

**Keywords:** mediastinal mass, robotic-assisted thoracoscopic surgery, perioperative outcome, body mass index, video-assisted thoracoscopic surgery

## Abstract

**Background:**

The effectiveness of robotic-assisted thoracoscopic surgery (RATS) for mediastinal masses has not been fully evaluated. This study aimed to compare the perioperative outcomes between RATS and video-assisted thoracoscopic surgery (VATS) for mediastinal masses, and then explore which group of people would benefit more from RATS.

**Methods:**

This retrospective study compared the perioperative outcomes of patients with mediastinal masses who underwent RATS and VATS from September 2018 to December 2021. Subgroup analysis were performed according to body mass index (BMI) ranges.

**Results:**

A total of 212 patients with mediastinal masses (106 RATS cases and 106 VATS cases) were included. Compared with the VATS group, the RATS group had a significantly reduced incidence of overall postoperative complications (5.7% vs. 14.2%, *p* = 0.039), complications of grade II or less (3.8% vs. 12.3%, *p* = 0.023), and pneumonia (2.8% vs. 9.4%, *p* = 0.045). Hospitalization costs were significantly higher in the RATS group (¥ 49350.0 vs. ¥ 32551.9, *p* < 0.001). There was no significant difference in operation duration, intraoperative estimated blood loss, postoperative chest tube drainage volume, NRS pain score, day of chest tube removal, complications of grade III or more, or in-hospital mortality rate (*p* > 0.05). Subgroup analysis indicated that the incidence of overall postoperative complications (3.1% vs. 15.2%, *p* = 0.017), complications of grade II or less (1.5% vs. 12.1%, *p* = 0.033) and postoperative length of stay (4 days vs. 4.5 days, *p* = 0.046) were significantly reduced in the RATS group for overweight and obese patients (BMI ≥ 24 kg/m^2^), while these differences became insignificant in the BMI < 24 kg/m^2^ subgroup.

**Conclusion:**

RATS could reduce the incidence of postoperative complications, shorten the postoperative length of stay and might be a more cost-effective surgical treatment for overweight and obese patients with mediastinal masses.

## Introduction

Mediastinal masses comprise a heterogeneous group of tumors, including thymomas, neurogenic tumors, teratomas, bronchogenic cysts, and thyroid tumors (1). Mediastinal tumors are located in various positions of the mediastinum and account for approximately 3% of thoracic diseases (2). Radical surgical resection remains the gold standard for diagnosis, treatment and staging of the majority of these tumors (3–5). The small space and complex structure of the mediastinum, surrounded by large blood vessels and important organs such as the heart, make this type of surgery a great challenge for thoracic surgeons (6). With the development of minimally invasive techniques, video-assisted thoracoscopic surgery (VATS) has been widely applied for mediastinum masses resection with satisfactory outcomes compared with traditional thoracotomy (7). As an emerging minimally invasive technique, robotic-assisted thoracoscopic surgery (RATS) has gradually become a prevalent surgical method for patients with mediastinal masses.

Since the first robotic-assisted thymectomy was reported by Yoshino et al. in 2001 (8), RATS has become increasingly used for the surgical treatment of mediastinal masses (9, 10). Compared with VATS, robotic-assisted systems can provide surgeons with many advantages, including naked eye three-dimensional (3D) imaging with 10–15 times magnification, 360° rotating mechanical arms with a reduction in hand-related tremors and better maneuverability, improved dexterity, and greater comfort (11). Although there has been a recent increase in the popularity and research on RATS, its effectiveness in mediastinal surgery remains controversial (12, 13). The majority of published studies comparing minimally invasive surgeries for mediastinal mass resection were performed mainly in small cohort and focused only on the treatment of thymoma or anterior mediastinal masses, providing limited evidence to determine which one is a more beneficial surgical approach. In addition, few studies have compared the efficacy of RATS and VATS in the treatment of mediastinal masses in different mediastinal locations. Currently, it is still controversial which minimally invasive approach is superior for the surgical treatment of mediastinal tumors.

The aim of this study was to compare the perioperative outcomes of patients with mediastinal masses who underwent RATS and VATS, and then determine which group of people would benefit more from RATS.

## Patients and methods

This retrospective study was approved by the institutional review board of the Qilu Hospital of Shandong University (registration number: KYLL-2020027), and all patients provided informed consent for the use of their clinical information.

### Patient selection

A prospectively maintained departmental database of Qilu Hospital of Shandong University was retrieved for patients who underwent a RATS or VATS for mediastinal mass from September 2018 to December 2021. The inclusion criteria were patients aged ≥18 years old who underwent mediastinal mass resection with detailed medical records. The exclusion criteria were: (I) patients aged <18 years old; (II) pulmonary resection with mediastinal mass resection; (III) thoracotomy; (IV) thymic cancer or thymoma with Masaoka-Koga stage greater than II; (V) patients with a history of myasthenia gravies or thoracic surgery; and (VI) incomplete perioperative data.

### Data collection and variable definitions

The following clinical data of enrolled patients were collected from the database of Qilu Hospital: age, sex, smoking history, body mass index (BMI), percentage of predicted value for forced expiratory volume in 1 s (FEV1% predicted), American Society of Anesthesiologists (ASA) score, operative approach (RATS or VATS), tumor location, operation duration, intraoperative estimated blood loss, postoperative drainage volume, day of chest tube removal, postoperative Numerical Rating Scale (NRS) pain score, postoperative complications, postoperative length of stay (POS), total cost of hospitalization, and pathological information. The choice of surgical approach mainly depends on the patients' acceptance of RATS. Based on good preoperative communication with the patients, the patients chose the surgical method independently. Tumor location was determined based on the three-division method of the mediastinum, and tumor size was defined as the maximum tumor diameter. Postoperative complications were classified according to the Clavien–Dindo classification, including pneumonia, chylothorax and arrhythmia. The volume of postoperative drainage was recorded by the nurse at 6:00 am every day after the operation. The NRS pain score was evaluated by the nurse at 24, 48, and 72 h after surgery and was defined as the postoperative day (POD) 1, 2, and 3 NRS score.

### Operative procedures

**Figure 1:**
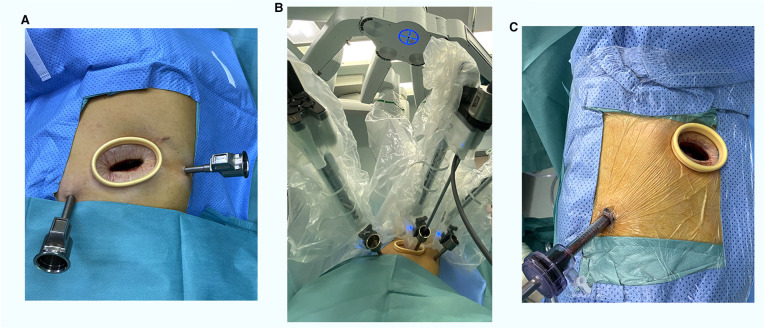
The incisions and ports placement of RATS (**A,B**) and VATS (**C**). RATS, robotic-assisted thoracoscopic surgery; VATS, video-assisted thoracoscopic surgery.

All of the surgeries were performed by 3 qualified surgeons in a single operation group. The patients in both groups underwent intravenous inhalation combined with anesthesia, and single-lumen tracheal intubation and occluder were used for single-lung ventilation. The patients with anterior mediastinal tumors were placed in a 30-degree semi-supine position with the ipsilateral axilla exposed, while lateral prone position was applied for patients with middle and posterior mediastinal tumors to reduce the interference of lung tissue. Right or left approach was selected according to the location of the tumor body, and right approach was mostly used for tumors located in the middle. VATS was performed using standard thoracoscopic techniques with two conventional incision operations for anterior mediastinal masses: one 3 cm auxiliary operative incision at the 2nd or 3rd intercostal space (ICS) on the anterior axillary line, and one camera port at the 5th ICS mid-axillary line. While uniport VATS was performed for middle and posterior mediastinal masses, and the port was set at the 5th ICS between the mid-axillary line and posterior axillary line. RATS was performed using the fourth-generation Da Vinci surgical system with a three-port approach. For patients whose tumor was located in the front mediastinum, the camera port was selected at the 5th ICS on the anterior axillary line, and two mechanical arm ports were set at the 5th ICS on the midclavicular line and approximately 2 cm posterior to the 6th ICS on the posterior axillary line, respectively. For those with tumors at the middle and posterior mediastinum, the camera port was selected at the 5th ICS on the anterior axillary line, and two mechanical arm ports were set at the 3th ICS on the anterior axillary line and the 7th ICS on the posterior axillary line, respectively. The position of the auxiliary operative incision was located at the 5th ICS between the anterior axillary line and mid-axillary line, and the interval between the three mechanical arms was approximately 6–8 cm. The incisions and ports placement of RATS and VATS are shown in Figure 1. The lesion resection was only performed if thymic cysts, lymphatic cysts, teratoma with intact capsule or other benign tumors were identified during the operation, and thymic tumors resection and total thymectomy were performed for patients whose preoperative clinical diagnosis did not exclude thymoma. One or two chest tubes were placed after the operation depending on surgeon performance.

### Postoperative management

All patients received postoperative analgesia with an analgesic pump, and the intravenous use of nonsteroidal anti-inflammatory drugs 3 times a day was applied for pain relief. The chest tube could be removed if there was no pneumonia, subcutaneous emphysema or pneumothorax with daily drainage less than 200 ml. All patients in this study were managed using an enhanced recovery after surgery program.

### Statistical analysis

Categorical variables were compared using the Pearson chi-squared test or Fisher's exact test. Normally distributed continuous variables are presented as the mean ± standard deviation (SD), and Student's *t* test was used for comparisons. For continuous variables that were not normally distributed, data are presented as the median (interquartile range [IQR]) and were compared by the Mann–Whitney U test between the groups. The test level between the 2 groups was set at *α* = 0.05 (bilateral), and a two-sided *p* < 0.05 was considered statistically significant. Subgroup analyses were performed for the perioperative outcomes according to BMI ranges. SPSS software v25.0 (SPSS Inc., Chicago, IL, USA) was used for further data analysis.

## Results

### Patient characteristics

Ultimately, a total of 212 patients with mediastinal masses (106 RATS patients and 106 VATS patients) were included for analysis in this study. The characteristics of the patients are presented in Table 1. Benign cyst (43.4%) was the most common histology followed by thymoma (25.9%), neurogenic tumor (12.3%), teratoma (6.1%), and thymic hyperplasia (4.2%). The patients who underwent VATS and RATS were comparable in age, sex, BMI, smoking history, FEV1% predicted, ASA score, mediastinal location, pathological type, and tumor size (*p* > 0.05).

**Table 1:** Clinicopathological characteristics of patients with mediastinal masses.

Characteristics	VATS (*n* = 106)	RATS (*n* = 106)	*p*
Age (years), median (IQR)	48 (39.75–56)	46 (33.75–57)	0.127
Sex, *n* (%)			1.000
Female	45 (42.5)	45 (42.5)	
Male	61 (57.5)	61 (57.5)	
BMI (kg/m^2^), median (IQR)	25.0 (22.5–27.4)	24.8 (22.9–26.9)	0.969
Smoking history, *n* (%)			0.730
Non-smoker	86 (81.1)	84 (79.2)	
Smoker	20 (18.9)	22 (20.8)	
FEV1% predicted, median (IQR)	99.9 (92.8–107.9)	100.4 (91.7–107.0)	0.909
ASA score, *n* (%)			0.571
I	26 (24.5)	28 (26.4)	
II	78 (73.6)	78 (73.6)	
III	2 (1.9)	0	
Mediastinal location, *n* (%)			0.388
Anterior	80 (75.5)	88 (83.0)	
Middle	4 (3.8)	3 (2.8)	
Posterior	22 (20.8)	15 (14.2)	
Pathological type, *n* (%)			0.479
Thymoma	23 (21.7)	32 (30.2)	
Thymic hyperplasia	5 (4.7)	4 (3.8)	
Benign cyst	53 (50.0)	39 (36.8)	
Neurogenic tumor	12 (11.3)	14 (13.2)	
Teratoma	6 (5.7)	7 (6.6)	
Other	7 (6.6)	10 (9.4)	
Tumor size (cm), median (IQR)	5.0 (3.5–7.0)	4.9 (3.5–6.3)	0.225

*IQR, interquartile range; RATS, robotic-assisted thoracoscopic surgery; VATS, video-assisted thoracoscopic surgery; BMI, body mass index; FEV1% predicted, percentage of predicted value for forced expiratory volume in 1 s; ASA, American Society of Anesthesiologists.*

### Perioperative outcomes

A comparison of the perioperative outcomes of the patients who underwent RATS or VATS is presented in Table 2. The incidence of overall postoperative complications (5.7% vs. 14.2%, *p* = 0.039), complications of grade II or less (3.8% vs. 12.3%, *p* = 0.023), and pneumonia (2.8% vs. 9.4%, *p* = 0.045) were significantly decreased in the RATS group. And hospitalization cost [¥ 49350.0 (IQR, 47938.7–51681.9) vs. ¥ 32551.9 (IQR, 29971.5–35555.3), *p* < 0.001] were significantly increased in the RATS group. However, there were no significant differences in operation duration operation duration [75 min (IQR, 60–95) vs. 75 min (IQR, 60–90), *p* = 0.329], intraoperative estimated blood loss [55 ml (IQR, 45–70) vs. 60 ml (IQR, 50–70), *p* = 0.113], the drainage volume on POD 1 [120 ml (IQR, 70–200) vs. 100 ml (IQR, 60–200), *p* = 0.117] and POD 2 [152.5 ml (IQR, 100–232.5) vs. 120 ml (IQR, 80–200), *p* = 0.086], NRS pain score on POD 1 [3 (IQR, 3–3) vs. 3 (IQR, 3–3), *p* = 0.088] and POD 2 [3 (IQR, 3–3) vs. 3 (IQR, 3–3), *p* = 0.690], day of chest tube removal [3 days (IQR, 3–4) vs. 3 days (IQR, 3–4), *p* = 0.533], POS [4 days (IQR, 3–5) vs. 4.5 days (IQR, 3–6), *p* = 0.062], complications of grade III or more (1.9% vs. 1.9%, *p* = 1.000), incidence of chylothorax (1.9% vs. 3.8%, *p *= 0.683) and arrhythmia (0.9% vs. 1.9%, *p *= 1.000), or in-hospital mortality rate (0.9% vs. 0, *p* = 1.000). There was no readmission and conversion to thoracotomy in either group.

**Table 2:** Perioperative outcomes of VATS and RATS for mediastinal masses.

Perioperative outcomes	RATS (*n* = 106)	VATS (*n* = 106)	*p*
Operation duration (min), median (IQR)	75 (60–95)	75 (60–90)	0.329
Estimated blood loss (ml), median (IQR)	55 (45–70)	60 (50–70)	0.113
Conversion to thoracotomy, *n* (%)	0	0	
Chest tube drainage (ml), median (IQR)			
POD 1	120 (70–200)	100 (60–200)	0.117
POD 2	152.5 (100–232.5)	120 (80–200)	0.086
Chest tube removal (d), median (IQR)	3 (3–4)	3 (3–4)	0.533
NRS score, median (IQR)			
POD 1	3 (3–3)	3 (3–3)	0.088
POD 2	3 (3–3)	3 (3–3)	0.690
Postoperative complications, *n* (%)	6 (5.7)	15 (14.2)	**0**.**039**
Severity grade of complications, *n* (%)			
Clavien-Dindo ≤ II	4 (3.8)	13 (12.3)	**0**.**023**
Clavien-Dindo ≥ III	2 (1.9)	2 (1.9)	1.000
Frequent complications, *n* (%)			
Pneumonia	3 (2.8)	10 (9.4)	**0**.**045**
Chylothorax	2 (1.9)	4 (3.8)	0.683
Arrhythmia	1 (0.9)	2 (1.9)	1.000
In-hospital mortality, *n* (%)	1 (0.9)	0	1.000
Readmission, *n* (%)	0	0	
POS (d), median (IQR)	4 (3–5)	4.5 (3–6)	0.062
Hospitalization cost (¥), median (IQR)	49350.0 (47938.7–51681.9)	32551.9 (29971.5–35555.3)	**<0.001**

*NRS, numerical rating scale; POD, postoperative day; POS, postoperative length of stay; IQR, interquartile range; RATS, robotic-assisted thoracoscopic surgery; VATS, video-assisted thoracoscopic surgery.*

*P values less than 0.05 are bolded.*

### Subgroup analysis

To explore which group of people would benefit more from RATS, a subgroup analysis was performed for the perioperative outcomes according to BMI ranges**.** The patients were divided into 2 groups based on their BMI: BMI < 24 kg/m^2^ and BMI ≥ 24 kg/m^2^, and the subgroup comparisons of perioperative outcomes between the RATS and VATS groups are presented in Table 3. Interestingly, we found that the incidence of overall postoperative complications (3.1% vs. 15.2%, *p* = 0.017), complications of grade II or less (1.5% vs. 12.1%, *p* = 0.033) and POS [4 days (IQR, 3–5) vs. 4.5 days (IQR, 4–6), *p* = 0.046] were significantly reduced in the RATS group for overweight and obese patients (BMI ≥ 24 kg/m^2^), while these differences became insignificant in the BMI < 24 kg/m^2^ subgroup. There was no significant difference in operation duration, intraoperative estimated blood loss, postoperative chest tube drainage volume, NRS pain score, day of chest tube removal, complications of grade III or more, or in-hospital mortality rate (*p* > 0.05).

**Table 3:** Perioperative outcomes of VATS and RATS for mediastinal masses in patients with different BMI ranges.

Characteristics	BMI < 24 kg/m^2^			BMI ≥ 24 kg/m^2^		
	RATS (*n* = 41)	VATS (*n* = 40)	*p*	RATS (*n* = 65)	VATS (*n* = 66)	*p*
Operation duration (min), median (IQR)	65 (60–90)	75 (60–90)	0.490	85 (65–100)	75 (60–90)	0.068
Estimated blood loss (ml), median (IQR)	50 (40–67.5)	57.5 (50–75)	0.144	55 (45–70)	60 (50–70)	0.395
Chest tube drainage (ml), median (IQR)						
POD 1	120 (80–215)	100 (42.5–200)	0.244	120 (65–200)	120 (60–195)	0.273
POD 2	160 (80–260)	115 (62.5–175)	0.060	150 (100–220)	160 (100–200)	0.542
Chest tube removal (d), median (IQR)	3 (3–4)	3 (2–4)	0.455	3 (3–4)	3.5 (3–4)	0.153
NRS score, median (IQR)						
POD 1	3 (3–3)	3 (3–3)	0.060	3 (3–3)	3 (3–3)	0.446
POD 2	3 (3–3)	3 (3–3)	0.750	3 (3–3)	3 (3–3)	0.441
Postoperative complications, *n* (%)	4 (9.8)	5 (12.5)	0.737	2 (3.1)	10 (15.2)	**0**.**017**
Severity grade of complications, *n* (%)						
Clavien-Dindo ≤ II	3 (7.3)	5 (12.5)	0.482	1 (1.5)	8 (12.1)	**0**.**033**
Clavien-Dindo ≥ III	1 (2.4)	0	1.000	1 (1.5)	2 (3.0)	1.000
Frequent complications, *n* (%)						
Pneumonia	2 (4.9)	5 (12.5)	0.264	1 (1.5)	5 (7.6)	0.208
Chylothorax	2 (4.9)	0	0.494	0	4 (6.1)	0.119
Arrhythmia	0	0		1 (1.5)	2 (3.0)	1.000
In-hospital mortality, *n* (%)	0	0		1 (1.0)	0	0.496
POS (d), median (IQR)	4 (3.5–5)	4.5 (3–6)	0.641	4 (3–5)	4.5 (4–6)	**0**.**046**
Hospitalization cost (¥), median (IQR)	49938.1 (47979.6-52752.0)	32501.0 (30019.8–35653.0)	**<0.001**	49191.1 (47841.9–50685.5)	32594.6 (29806.6–35458.8)	**<0.001**

*BMI, body mass index; NRS, numerical rating scale; POD, postoperative day; POS, postoperative length of stay; IQR, interquartile range; RATS, robotic-assisted thoracoscopic surgery; VATS, video-assisted thoracoscopic surgery.*

*P values less than 0.05 are bolded.*

## Discussion

In recent years, there has been a remarkable increase in the popularity of RATS, but its role and potential advantages as a surgical treatment for mediastinal masses have not been well illustrated. This retrospective study compared the perioperative outcomes between RATS and VATS for mediastinal masses, and aimed to explore which group of people would benefit more from RATS. We have performed subgroup analyses according to age, BMI and tumor location, and found that the advantages of RATS might be more obvious in overweight and obese people. The results of our study indicated that RATS might have potential advantages compared with VATS in terms of reducing the incidence of postoperative complications and shortening POS for overweight and obese patients with mediastinal masses, while RATS and VATS have comparable perioperative outcomes in patients with a BMI less than 24 kg/m^2^. It is the first study to explore the advantages and disadvantages of RATS for patients with mediastinal masses in different BMI ranges.

Mediastinal masses are mainly treated by surgical resection in clinical practice, and some patients require adjuvant postoperative radiotherapy and chemotherapy (14). At present, VATS is the mainstream surgical method for mediastinal tumors. The incision of VATS is small and located in the intercostal space, which well protects the bony thorax and reduces the damage to the body to a certain extent (4, 7). RATS, as an emerging minimally invasive surgical approach, has become increasingly used for the surgical treatment of mediastinal masses with good clinical efficacy and safety since the first application reported by Yoshino et al. in 2001 (8, 9). The naked 3D visualization and better maneuverability provided by the surgical robotic system allow the surgeons to dissect the tissues, vessels and nerves surrounding the tumor more clearly. In addition, RATS has revealed unique superiority over VATS while dealing with locally invasive diseases and tumors in narrow space (15).

Several studies have been conducted to compare the safety and efficacy of RATS and VATS as surgical treatments for mediastinal masses. Zeng et al. conducted a retrospective study to identify the feasibility of RATS compared with VATS in the resection of mediastinal lesions (16). The results showed that RATS had non-inferior postoperative outcomes and better intraoperative safety with a lower incidence rate of unplanned thoracotomy than the VATS approach. Christine et al. retrospectively compared the outcomes of mediastinal tumor resection with RATS and VATS, and found that RATS resection was associated with fewer conversion, fewer positive margins, shorter length of stay and less composite adverse events (17). In this study, we found that RATS might provide better safety due to a significantly reduced incidence of postoperative complications. However, total hospitalization costs with RATS were significantly higher than those with VATS. Therefore, it is necessary to consider cost performance when choosing RATS as an alternative surgical treatment for mediastinal masses.

A highlight of this study is the comparison of perioperative outcomes between RATS and VATS in patients with different BMI ranges, aiming at exploring which group of people would benefit more from RATS. The results of subgroup analysis demonstrated that the incidence of postoperative complications and POS was significantly reduced in the RATS group for overweight and obese patients (BMI ≥ 24 kg/m^2^). However, for patients with BMI < 24 kg/m^2^, RATS did not achieve better perioperative outcomes than VATS but had a significantly increased expense, indicating it might be not cost-effective to select RATS for these patients with mediastinal masses. In recent years, there was a significant increase in the number of obese and overweight patients with mediastinal tumors. Thoracic surgeons would encounter great challenges when operating on overweight and obese patients due to increased internal fat, limited movements of instruments, deeper thoracic cavity and their well-known poor outcomes (18). In this study, we found that RAL might achieve better perioperative outcomes for overweight and obese patients, and RATS might be a more beneficial surgical treatment for overweight and obese patients with mediastinal masses.

This study has several limitations that should be considered. First, the single-center retrospective nature of this study makes it less persuasive than a multicenter prospective randomized controlled trial. Second, some outcomes, such as intraoperative estimated blood loss, and operative duration, are closely related not only to the surgical approaches but also to the performance of the surgeon. It is difficult to untangle the effects of the two on the outcomes. Third, the fourth-generation DaVinci robot surgical system is typically applied for RATS, thus further investigation is needed to determine whether our results can be generalized to other centers where other robotic systems may be more common. Finally, the long-term prognostic outcomes were not compared because the follow-up period has not been reached, which need to be further investigated in future studies.

## Conclusion

RATS could reduce the incidence of postoperative complications, shorten the postoperative length of stay and might be a more cost-effective surgical treatment for overweight and obese patients with mediastinal masses.

## Data Availability

The original contributions presented in the study are included in the article/Suplementary Material, further inquiries can be directed to the corresponding author/s.

## References

[1] Carter BW Benveniste MF Madan R Godoy MC de Groot PM Truong MT ITMIG Classification of mediastinal compartments and multidisciplinary approach to mediastinal masses. Radiographics. (2017) 37(2):413–36. 10.1148/rg.2017160095 28129068

[2] Aroor AR Prakasha SR Seshadri S ST Raghuraj U. A study of clinical characteristicsof mediastinal mass. J Clin Diagn Res. (2014) 8(2):77–80. 10.7860/JCDR/2014/7622.4013 PMC3972605 24701488

[3] Hwang SK Park SI Kim YH Kim HR Choi SH Kim DK. Clinical results of surgical resection of mediastinal teratoma: efficacy of video-assisted thoracic surgery. Surg Endosc. (2016) 30(9):4065–8. 10.1007/s00464-015-4721-9 26694183

[4] Guo C Mei J Liu C Deng S Pu Q Lin F Video-assisted thoracic surgery compared with posterolateral thoracotomy for mediastinal bronchogenic cysts in adult patients. J Thorac Dis. (2016) 8(9):2504–11. 10.21037/jtd.2016.08.29 27747002 PMC5059344

[5] Scorsetti M Leo F Trama A D’Angelillo R Serpico D Macerelli M Thymoma and thymic carcinomas. Crit Rev Oncol Hematol. (2016) 99:332–50. 10.1016/j.critrevonc.2016.01.012 26818050

[6] Ronson RS Duarte I Miller JI. Embryology and surgical anatomy of the mediastinum with clinical implications. Surg Clin North Am. (2000) 80(1):157–69.. 10.1016/S0039-6109(05)70400-X 10685147

[7] Hess NR Sarkaria IS Pennathur A Levy RM Christie NA Luketich JD. Minimally invasive versus open thymectomy: a systematic review of surgical techniques, patient demographics, and perioperative outcomes. Ann Cardiothorac Surg. (2016) 5(1):1–9. 10.3978/j.issn.2225-319X.2016.01.01 26904425 PMC4740099

[8] Yoshino I Hashizume M Shimada M Tomikawa M Sugimachi K. Video-assisted thoracoscopic extirpation of a posterior mediastinal mass using the da Vinci computer enhanced surgical system. Ann Thorac Surg. (2002) 74(4):1235–7. 10.1016/S0003-4975(02)03820-1 12400778

[9] Chen K Zhang X Jin R Xiang J Han D Zhang Y Robot-assisted thoracoscopic surgery for mediastinal masses: a single-institution experience. J Thorac Dis. (2020) 12(2):105–13. 10.21037/jtd.2019.08.105 32190360 PMC7061195

[10] Li H Li J Huang J Yang Y Luo Q. Robotic-assisted mediastinal surgery: the first Chinese series of 167 consecutive cases. J Thorac Dis. (2018) 10(5):2876–80. 10.21037/jtd.2018.04.138 29997952 PMC6006102

[11] Buentzel J Heinz J Hinterthaner M Schöndube FA Straube C Roever C Robotic versus thoracoscopic thymectomy: the current evidence. TInt J Med Robot. (2017) 13(4):e1847. 10.1002/rcs.1847 28660682

[12] Marulli G Comacchio GM Schiavon M Rebusso A Mammana M Zampieri D Comparing robotic and trans-sternal thymectomy for early-stage thymoma: a propensity score-matching study. Eur J Cardio-Thorac Surg. (2018) 54(3):579–84. 10.1093/ejcts/ezy075 29547970

[13] Şehitogullari A Nasır A Anbar R Erdem K Bilgin C. Comparison of perioperative outcomes of videothoracoscopy and robotic surgical techniques in thymoma. Asian J Surg. (2020) 43(1):244–50. 10.1016/j.asjsur.2019.04.005 31047770

[14] Carter BW Marom EM Detterbeck FC. Approaching the patient with an anterior mediastinal mass: a guide for clinicians. J Thorac Oncol. (2014) 9(9 Suppl 2):S102–9. 10.1097/JTO.0000000000000294 25396306

[15] Yoshino I Hashizume M Shimada M Tomikawa M Tomiyasu M Suemitsu R Thoracoscopic thymomectomy with the da Vinci computer-enhanced surgical system. J Thorac Cardiovasc Surg. (2001) 122(4):783–5. 10.1067/mtc.2001.115231 11581613

[16] Zeng L Wang W Han J Zhu L Zhao J Tu Z. Uniportal video-assisted thoracoscopic surgery and robot-assisted thoracoscopic surgery are feasible approaches with potential advantages in minimally invasive mediastinal lesions resection. Gland Surg. (2021) 10(1):101–11. 10.21037/gs-20-536 33633967 PMC7882349

[17] Alvarado CE Worrell SG Bachman KC Jiang B Janko M Gray KE Robotic approach has improved outcomes for minimally invasive resection of mediastinal tumors. Ann Thorac Surg. (2022) 113(6):1853–8. 10.1016/j.athoracsur.2021.05.090 34217691

[18] Casiraghi M Sedda G Diotti C Mariolo AV Galetta D Tessitore A Postoperative outcomes of robotic-assisted lobectomy in obese patients with non-small-cell lung cancer. Interact Cardiovasc Thorac Surg. (2020) 30(3):359–65. 10.1093/icvts/ivz273 31755924

